# Incidence, causes, severity and treatment of throat discomfort: a four-region online questionnaire survey

**DOI:** 10.1186/1472-6815-12-9

**Published:** 2012-08-10

**Authors:** Dilys Addey, Adrian Shephard

**Affiliations:** 1 Addey Associates, 21 Stanstead Avenue, Tollerton, Nottingham, NG12 4EA, UK; 2 Reckitt Benckiser Group plc, 103-105 Bath Road, Slough, SL1 3UH, UK

**Keywords:** Sore throat, Throat discomfort, Online survey

## Abstract

**Background:**

Acute sore throat is commonly associated with viral infections. Consumers typically rely on over-the-counter treatments and other remedies to treat symptoms; however, limited information is available regarding consumer perceptions of sore throat or treatment needs. The aim of this study was to investigate perceptions of throat discomfort and how these influence attitudes and consumer behaviour with regard to treatment.

**Methods:**

Online consumer surveys were completed by participants invited by email between 2003 and 2004 in four markets: the UK, France, Poland, and Malaysia. The questionnaire consisted of 24 questions that covered key issues surrounding throat discomfort including incidence in the past 12 months, causes, severity, effects on functionality and quality of life, actions taken to relieve throat discomfort, the efficacy of these approaches and the reasons behind using specific products.

**Results:**

In total, 6465 men and women aged ≥18 years were surveyed, identifying 3514 participants who had suffered throat discomfort/irritation in the past 12 months (response rate of 54%). These participants completed the full survey. The breakdown of throat discomfort sufferers was: UK, 912; France, 899; Poland, 871; Malaysia, 832. A high proportion of respondents experienced one or more instances of throat discomfort in the previous 12 months, with an overall incidence of 54%. Infections including the common cold/influenza and other bacteria/viruses were commonly perceived causes of throat discomfort (72% and 46%, respectively). Physical and environmental factors were also perceived to be causative, including airborne pollution (28%), smoking (23%), and air conditioning (31%). Symptoms perceived to be caused by an infection were associated with a higher degree of suffering (mean degree of suffering for bacteria/virus and common cold/influenza; 3.4 and 3.0, respectively). Medicinal products were used for all perceived causes, but more commonly for sore throats thought to be caused by infections. Cold drinks were used more often for symptoms thought to be due to physical and environmental causes.

**Conclusions:**

Not all throat discomfort is the same, as demonstrated by the range of perceived causes and the emotional and physical symptoms experienced. Patient expectations regarding treatment of throat discomfort differs and treatments should be tailored by pharmacists to suit the cause.

## Background

Acute sore throat, which typically describes self-limiting pharyngitis, tonsillitis, and laryngitis, is one of the most common complaints that results in patients presenting to their general practitioner (GP) or pharmacist; however, most people with sore throat will not seek medical help [1]. Without effective treatment, such throat discomfort can significantly impact on the health-related quality of life of sufferers. Indeed, in one study of patients with acute sore throat caused by upper respiratory tract infection, simple daily activities such as swallowing, talking, eating, sleeping, working, and concentrating were rated as being impaired [2].

It has been estimated that 50–95% of sore throats in adults and 70% in children are caused by infection with respiratory viruses [3,4]. Bacterial infection is the cause of symptoms in only 20% of cases of throat discomfort [3]. A throat may not always be described as sore, with the terms ‘throat irritation’ or ‘discomfort’ also being used, and soreness can be triggered by causes other than infection such as pollution, allergens, and dry air [5-7]. Also, evidence suggests that environmental factors, such as cold temperature and low humidity, also play a significant role in the development of respiratory tract infections including not only pharyngitis (i.e. throat discomfort/irritation), but also common colds – themselves a cause of throat discomfort [8,9]. Therefore, the occurrence of throat discomfort may be more common than previously reported and may contribute to ‘sickness behaviour’ [10].

As the majority of sore throats are not of bacterial origin, the National Institute for Health and Clinical Excellence (NICE) and other international health authorities, recommend that antibiotics should not be used for primary treatment [11,12]. The need for and prescription of antibiotics are subject to national differences [13] and delaying or non-prescription of antibiotics also reduces the financial burden on health authorities [12]. Most cases of sore throat can be managed by patients using over-the-counter (OTC) treatments and other remedies as a means of controlling their symptoms [14]. However, to date, there is limited information available regarding consumers’ perceptions of throat discomfort or their treatment needs.

The aim of the current study was to investigate the incidence, causes, and degree of suffering associated with throat discomfort across four markets, and to better understand how these influence the attitudes and behaviour of consumers with regard to OTC therapies and other management approaches.

## Methods

To gain an overview of consumer needs across a disparate global population, online consumer surveys were completed by participants between 2003 and 2004 in four markets: the UK, France, Poland, and Malaysia. These countries were chosen as it was felt that they were broadly representative of different markets; the UK being an established general sales list (GSL) market where products can be purchased without the supervision of a pharmacist; France an established pharmacy-based market where products are purchased in a pharmacy only; Poland an emerging and pharmacy-based market; and Malaysia an emerging Asian market. The research was conducted in accordance with the Market Research Society Code of Conduct and the Data Protection Act of 1998.

### Participant recruitment

Fieldwork was conducted using the National Family Opinion (NFO) WorldGroup's On-Line Access Panel in the UK (June 2003), Ciao's On-Line Access Panels in France (August/September 2003) and Poland (November 2003), and the Taylor Nelson Sofres (TNS) On-Line Panel in Malaysia (July/August 2004). NFO/TNS have a global network of online managed access panels spanning North America, Europe and Asia Pacific.

Invitations to complete the questionnaire were emailed to a sample of online panellists in the relevant countries who agreed to participate in the market research. Respondents were provided with a link to the survey. The survey was open for a limited time period and then closed a few days later when the required sample profile had been achieved. Questionnaire filters and data checks were automatically applied during the course of the self-completion interview. Panellists were incentivised at the usual NFO agency rate at the time of £20.

### Population sampling

Respondent samples were planned to be representative of total populations of each country assessed with regard to key demographic variables. Market research agencies in different countries use different demographic variables, for instance, social class as a variable is only relevant in the UK.

Men and women aged ≥18 years who had suffered from throat irritation or throat discomfort in the past 12 months were surveyed. A tally of non-sufferers was kept to identify incidence of suffering during the past 12 months. The sample population included a range of key variables such as sex and age as well as social class and household size for the UK, income for France and Malaysia, and locality for Poland and Malaysia. A total base of 800 respondents per country was recommended as a minimum number to give a robust sample size and allow analysis of attitudes and behaviours among different sub-groups.

### Questionnaire development

The questionnaire was developed by NFO in English, and was then translated into the individual languages (see Additional file 1 for the questionnaire in English). The questionnaire was then back translated as a double check. The questionnaire consisted of 24 questions that covered key issues around incidence; causes (including the five most frequent); severity of throat discomfort (rated on a scale from 1–5: 1, very mild; 5, very severe); how the throat feels during periods of discomfort (dry, hurts to swallow, irritated, scratchy, husky, painful to talk, tickly, inflamed, burning, swollen, and swallowed glass/wire); effects of throat discomfort on consumer functionality and quality of life, including the emotional impact; general attitudes towards the illness; actions taken to relieve throat discomfort and the efficacy of these approaches; and reasons behind the use of specific products.

### Data analysis

Data were analyzed in the first instance on a per country basis and are presented as a mean value. To determine the frequency of suffering, respondents were first asked to select from a list the five most frequent causes of past cases of throat irritation or discomfort. Respondents were then asked to state how frequently they suffered from symptoms as a result of each of the causes stated during the previous 12 months. These data were used to calculate the mean frequency of suffering for each cause of throat discomfort. The severity of suffering was determined by asking respondents to rate, on a scale of 1–5, the degree of suffering associated with the cause of their throat discomfort. A mean of the degree of suffering attributed to each of the individual causes was then calculated. Respondents were also asked to select from a list, the course of action they took, as well as a descriptor of how their throat felt, during each of the stated causes of throat discomfort. Mean values were calculated for the proportion of respondents who selected each of the given choices.

To aid in the overall analysis, data were also pooled and the mean value from each of the four countries was used to calculate an overall mean for the entire study population.

## Results

### Demographic data

A total of 3514 participants were included in the analysis: 912 from the UK, 899 from France, 871 from Poland, and 832 from Malaysia (response rate of 54%). The total number of respondents surveyed from each country was 1940 from the UK, 1954 from France, 968 from Poland, and 1603 from Malaysia. Overall, the baseline demographics of participants were comparable across each of the countries included with regard to age and sex (Table 1).

**Table 1:** Demographics of respondents included in the sample population across the four countries assessed

	**UK (n=912)**	**France (n=899)**	**Poland (n=871)**	**Malaysia (n=832)**
**Age (years)**	18–34: 40%	18–34: 40%	18–24: 22%	18–24: 38%
	35–54: 41%	35–54: 40%	25–40: 43%	25–30: 36%
	≥55: 19%	≥55: 20%	≥41: 42%	31–40: 22%
				≥41: 5%
**Sex**				
Male	47%	47%	44%	50%
Female	53%	53%	56%	50%
				
**Social class**	ABC1: 63%	NR	NR	Employed: 73%
	C2DE: 37%			Unemployed: 5%
				Student: 22%
**Income**	NR	Low: 36%	NR	Up to RM1999: 24%
		Medium: 25%		RM2000–2999: 18%
		High: 19%		RM3000–4999: 22%
		Refused to answer: 20%		RM5000+: 17%
				Refused to answer: 19%
**Household size**				
One member	14%	NR	NR	NR
Two members	33%			
Three members	21%			
Four members	21%			
Five or more	10%			
				
**Locality**	NR	Urban: 58%	Urban: 77%	City: 67%
		Rural: 42%	Rural: 23%	Suburbs: 22%
				Small town: 9%
				Rural: 2%

NR, not recorded; RM, Malaysian ringgit (monthly).

### Frequency of suffering

Across the four markets assessed, a high proportion of respondents had experienced one or more instances of throat discomfort in the previous 12 months, with an overall incidence of 3514/6465 (54%) participants. This included 47% (428/912) of respondents from the UK, 46% (413/899) from France, and 52% (433/832) from Malaysia. In Poland, 90% of respondents (784/871) suffered from throat discomfort in the 12 months prior to the survey.

### Range of perceived causes and severity of suffering

Respondents across all countries considered the common cold/influenza to be responsible for their throat discomfort in 72% of cases within the past 12 months (Table 2). This was followed by other bacteria/viruses, sudden changes in temperature, hot and dry indoor conditions, and dust and outdoor conditions.

**Table 2:** Perceived causes of cases of throat discomfort ever suffered across (proportion of patients, %)

	**UK**	**France**	**Poland**	**Malaysia**	Overall*
Common cold/influenza	76	58	84	69	**72**
Other bacteria/virus	53	31	53	45	**46**
Talking/shouting	35	21	33	28	**30**
Hot and dry indoor conditions	33	24	37	42	**34**
Dust and outdoor conditions	26	35	24	52	**34**
Hay fever	25	19	14	10	**17**
Air conditioning	23	45	23	32	**31**
Excess alcohol	22	10	11	3	**12**
Smoking	21	30	23	17	**23**
Passive smoking	21	19	11	21	**18**
Airborne pollution	19	32	19	41	**28**
Sudden change in temperature	16	58	51	45	**43**
Snoring	16	12	8	7	**11**
Specific allergy	6	11	9	13	**10**
Other	4	3	3	0	**3**

*‘Overall’ represents a pooled analysis of mean data from all four countries assessed. Respondents were allowed to select several causes.

Data are from the four markets assessed in the past 12 months.

Interestingly, among Malaysian respondents, dust and outdoor conditions, airborne pollution, and hot and dry indoor conditions were considered as causative more commonly than those from any other country assessed (Table 2). Indeed, when asked how frequently they experienced each specific cause of throat irritation, 14% of respondents in Malaysia claimed to suffer more often than once weekly from throat discomfort as a result of hot and dry conditions (data not shown). Temperature changes may also have led to 12% of respondents experiencing throat discomfort more often than once weekly (data not shown).

A pooled analysis demonstrated that snoring, passive smoking, smoking, specific allergy, air conditioning, and dust/other outdoor conditions were considered to be responsible for the highest degree of annual suffering across all four countries (Table 3). Common cold/influenza or infections with other bacteria/viruses were thought to be causative in an average of four and three occasions during the past 12 months, respectively.

**Table 3:** Perceived causes of throat discomfort by country

	Mean annual frequency of suffering by cause*				
	**UK**	**France**	**Poland**	**Malaysia**	**Overall**^**†**^
Common cold/influenza	3	3	3	6	**4**
Other bacteria/virus	3	3	2	4	**3**
Talking/shouting	8	11	10	10	**10**
Hot/dry indoor conditions	13	7	13	10	**11**
Dust/other outdoor conditions	14	11	11	12	**12**
Air conditioning	13	9	8	18	**12**
Excess alcohol	11	7	9	15	**11**
Smoking	13	22	17	18	**18**
Passive smoking	17	18	19	20	**19**
Hay fever	18	8	10	3	**10**
Specific allergy	14	13	18	12	**14**
Airborne pollution	11	11	8	9	**10**
Sudden temperature change	10	6	6	10	**8**
Snoring	25	29	15	13	**21**

*Mean of the number of occasions that throat irritation by the stated cause was suffered in the preceding 12 months.

^**†**^‘Overall’ represents a pooled analysis of mean data from all four countries assessed.

Across the four countries included, infection with bacteria/viruses was classified as causing the highest degree of suffering (mean score 3.4), followed by common cold/influenza infection (mean score 3.0), specific allergy (mean score 2.8), and hay fever (mean score 2.7). Other causes of sore throat that led to a high degree of suffering were passive smoking (mean score 2.7), sudden change in temperature (mean score 2.7), and air conditioning and talking/shouting (mean score 2.6). The least degree of suffering was caused by throat irritation associated with snoring (mean score 2.3).

### Range of physical and emotional symptoms experienced during periods of throat discomfort

**Figure 1:**
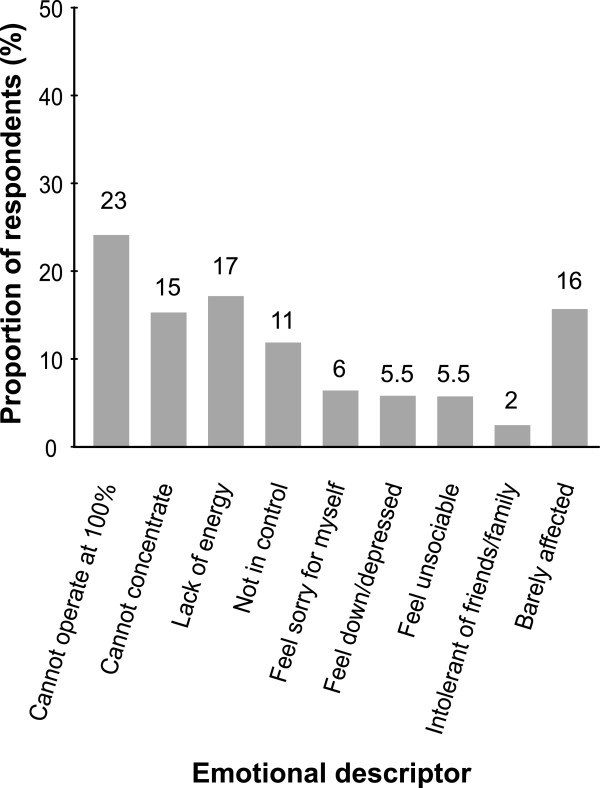
** Descriptors used by respondents to describe their emotional feelings during periods of throat discomfort.** Data are from a pooled analysis of mean data from all four countries assessed.

The overall descriptors used by respondents to indicate how their throat felt during periods of discomfort were similar across the countries assessed and differed depending on the perceived cause of the throat discomfort (Table 4). For example, all respondents who suffered from throat discomfort as a result of a perceived cause of infection included the descriptors ‘hurts to swallow’ and ‘irritated’. In contrast, throat discomfort as a result of environmental factors was consistently described as ‘dry’, ‘irritated’, and ‘tickly’. Those with throat discomfort caused by sudden changes in temperature described their symptoms as ‘hurts to swallow’ and ‘irritated’ in all cases. ‘Husky’ was attributed to throat discomfort caused by snoring, passive smoking, smoking, and talking/shouting. Based on these descriptors, throat discomfort perceived to be caused by infection is thought by respondents to be most severe. This is in agreement with the highest perceived degree of suffering being associated with throat discomfort thought to be caused by infection with bacteria/viruses. Across all countries, most sufferers felt ‘frustration that they cannot operate at 100%’ when suffering with throat discomfort (Figure 1). Other commonly experienced emotions were ‘lack of energy’, ‘cannot concentrate’, and ‘not in control’.

**Table 4:** Descriptors consistently used by respondents to indicate how their throat feels during periods of discomfort

**Cause of throat discomfort**	**Descriptors**
Common cold/influenza	Hurts to swallow
	Irritated
	Dry
Other bacteria/virus	Hurts to swallow
	Inflamed
	Irritated
Talking/shouting	Painful to talk
	Husky
	Dry
Hot and dry indoor conditions	Dry
	Tickly
	Irritated
Dust or other outdoor conditions	Dry
	Tickly
	Irritated
Air conditioning	Dry
	Tickly
	Irritated
Passive smoking or smoking	Dry
	Irritated
	Tickly
	Husky
Snoring	Dry
	Husky
	Irritated
Sudden changes in temperature	Hurts to swallow
	Irritated
	Tickly

### General attitude to illness

**Figure 2:**
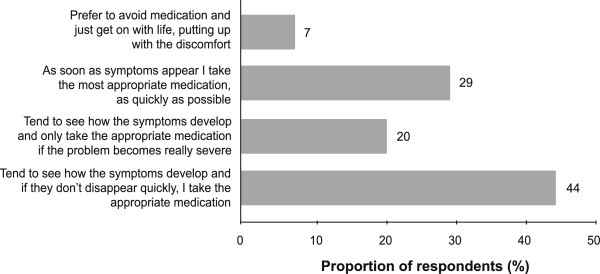
** General attitudes to throat discomfort and medication.** Data shown are from a pooled analysis of mean responses from across the four markets assessed.

Overall, responses regarding general attitudes towards illness from across the four countries and between sexes were similar, with the majority of participants stating that they ‘tend to see how symptoms develop and if they don’t disappear quickly, I take the appropriate medication’ (Figure 2). Only a small proportion of respondents ‘prefer to avoid medication and just get on with life, putting up with the discomfort’.

Interestingly, there was a difference between countries in the proportion of respondents who selected ‘as soon as symptoms appear, I take the most appropriate medication as quickly as possible’. This was chosen by 17% and 26% of respondents in the UK and France, respectively, compared with 44% in Poland and 30% in Malaysia (data not shown).

### Action taken by cause

Data were once again similar across the four markets (Table 5) when participants were asked to select the course of action they take when they suffer throat discomfort. Respondents suffering from throat discomfort caused by perceived bacterial/viral infection or cold/influenza were most likely to use medicated products which contain a medicinal substance or active ingredient, hot drinks, throat sweets, or consult a GP. Those suffering from hay fever or allergy most frequently consulted a GP or took medicated products. There was also a tendency for respondents who suffered a higher degree of perceived discomfort and those who had suffered for longer periods of time (>4 days) to use medicated treatments (data not shown).

**Table 5:** Action taken for throat discomfort by specific cause and severity of discomfort (% of respondents)

	**Cold/ influenza**	**Bacteria/virus**	**Hay fever**	**Hot/dry**	**Dust**	**Pollution**	**Talking/shouting**	**Snoring**	**Air-con**	**Temp change**	**Allergy**	**Alcohol**	**Smoking**	**Passive smoking**
Medicated products	62	54	31	32	32	33	38	22	34	43	34	20	32	28
Hot drinks	43	35	29	30	25	26	25	19	31	36	25	21	24	22
Vitamins	40	36	24	16	20	25	15	13	15	25	19	12	15	13
Throat sweets	31	35	26	22	21	20	27	7	23	25	24	14	19	17
Consulted GP	31	49	33	9	14	15	9	11	11	17	34	6	8	8
Herbal remedies	17	17	16	13	12	15	13	8	10	14	14	9	9	11
Confectionery	11	7	10	13	13	12	14	8	12	11	10	11	14	15
Chewing gum	11	9	12	26	15	1	13	10	18	15	10	23	18	16
Cold drinks	10	8	12	25	19	16	21	22	19	16	14	34	21	21
**No treatment sought**	**3**	**3**	**11**	**10**	**18**	**18**	**16**	**33**	**14**	**10**	**11**	**17**	**19**	**25**
**Severity of discomfort**	**3.0**	**3.4**	**2.7**	**2.5**	**2.4**	**2.4**	**2.6**	**2.3**	**2.6**	**2.7**	**2.8**	**2.4**	**2.5**	**2.7**

Data is from a pooled analysis of mean data from all four countries assessed.

Respondents with throat discomfort perceived to be caused by factors other than infection differed with regard to treatment depending on the exact cause of their symptoms (Table 5). For example, those who suffered from throat discomfort as a result of hot and dry conditions, dust, air conditioning, or temperature changes were more likely to use medicated products and hot and/or cold drinks as a means of relief. In contrast, symptoms caused by talking/shouting were primarily treated with medicated products or throat sweets. There were also a number of respondents who did not treat their symptoms; primarily those with throat irritation caused by passive smoking, smoking, alcohol, or snoring.

The medicated product most widely used in the UK was Strepsils® (amylmetacresol/2,4-dichlorobenzyl alcohol), with Lockets® (menthol/eucalyptol) and Halls Soothers® (hexylresorcinol/benzalkonium chloride solution) the most commonly bought throat sweets. The two most widely bought medicated products in France were Solutricine® (biclotymol) and Lysopaine® (cetylpyridinium/lysozyme hydrochloride); and in Poland were Neo Angin® (2,4-Dichlorobenzyl alcohol/menthol/pentyl-m-cresol) and Cholinex® (choline salicylate). In Malaysia, respondents tended to select Strepsils® and Fisherman’s Friend® (menthol) for sore throat or throat discomfort, with Halls® (menthol) and Ricola® (burnet/elder blossom/speedwell/peppermint/sage/marshmallow/thyme/lady’s mantle/horehound/plantain/cowslip/yarrow/mallow) selected more for breath freshening, loss of voice or simply as a sweet (data not shown).

## Discussion

Overall, the results of this consumer study demonstrate that over half of the respondents experienced throat discomfort or irritation in the past 12 months. The common cold/influenza was perceived to be the most common cause of throat discomfort across the countries surveyed although environmental and physical factors were also major perceived causes. Infections with bacteria/viruses and the common cold/influenza were classified as causing the highest degree of suffering across the countries. The descriptors used by respondents to indicate how their throat felt were similar across the countries and were dependent on the perceived cause of throat discomfort. The emotional impact and general attitude towards illness were similar across all countries as was the action taken in relation to the cause.

These data demonstrate that, in the mind of the consumer, throat irritation and discomfort may have a variety of perceived causes that can be broadly divided into three categories: the first category being caused by infections (e.g. caused by cold/influenza, bacterial/viral infection), the second being caused by environmental factors (e.g. allergy/hay fever, passive smoking, pollution, temperature, etc.), and the third a result of self-induced/physical factors (e.g. smoking, drinking, shouting, etc.).

Although infection was the most commonly reported cause of throat discomfort on an individual basis, when environmental factors were pooled these led to a higher overall frequency of illness. For example, respondents in Malaysia (a tropical country) reported that a high frequency of symptoms were caused by pollution, dust and outdoor conditions, and sudden changes in temperature, which could be linked with more time spent outdoors in a drier environment. This finding indicates that there is a degree of seasonality with regard to the perceived cause of throat discomfort: infection and allergy, such as hay fever, may be regarded as seasonal causes, whereas environmental causes may be regarded as non-seasonal.

The high incidence of throat discomfort in Poland was likely to be a result of seasonality, as the survey was conducted during November when the weather would have been colder and as such, sore throat may have been at the forefront of respondents’ minds, rather than indicating a higher incidence of throat discomfort per se. In Poland there can also be a large contrast between overheated public places, offices and shops, and the cold outdoors. Throat discomfort due to air conditioning is more frequently mentioned in France, where usage is higher due to seasonal high temperatures. Pollution is also frequently mentioned in Malaysia and France, which may be linked to pollution levels in Kuala Lumpur and Paris.

When asked about their treatment approach to illness and medication, most respondents stated that they ‘tend to see how symptoms develop and if they don’t disappear quickly, I take the appropriate medication’; although in all countries there were some respondents that would take appropriate medication as soon as symptoms appeared. This does indicate that there are differences in treatment approaches between consumers when it comes to their health. It has been previously reported that most people with sore throat do not seek medical help [1]. The treatment that was selected tended to differ depending on the perceived cause of the throat discomfort. Those with symptoms attributed to bacterial/viral infection or cold/influenza were most likely to use medicated products, hot drinks, throat sweets, or to consult a GP, although medical consultations are sought for other perceived causes. Symptoms that were a result of other causes were treated with a range of remedies including hot and/or cold drinks for those caused by hot and dry conditions, dust, air conditioning, or temperature changes; medicated products or throat sweets were generally used for irritation resulting from talking/shouting. Medicated lozenges were taken across multiple perceived causes of throat discomfort and across all countries; however, medicated lozenges may not always be appropriate for non-infectious causes of sore throat as they may contain local disinfectants to specifically target infectious agents.

The approach of respondents to treatment reflected the severity and perceived cause of their throat discomfort and irritation. Almost half of respondents waited a short time before seeking treatment, but around one-third had a treat early mentality, seeking treatment immediately upon experiencing symptoms. Throat discomfort that was rated as being most severe (i.e. that caused by infection or allergy) was typically treated using medicated products. More severe symptoms caused by environmental factors were treated using hot/cold drinks or confectionery. The use of products with a sensorial effect, i.e. warming or cooling, indicates specific needs from consumers. Those sore throats that were considered least severe were treated with a wider range of approaches.

Based on the descriptors and severity scores provided by respondents, throat discomfort and irritation caused by environmental factors was considered only slightly less severe than that caused by infection. Also, environmental and physical causes of throat discomfort affected respondents throughout the year and the ongoing and cumulative impact of such suffering may be significant. Furthermore, all types of throat irritation were reported to have an impact on the ability of respondents to function normally, except in 16% of cases. Responses were similar across all the countries assessed and in some cases the effect was so severe that respondents claimed to feel down/depressed or unsociable, demonstrating an emotional impact.

Based on these data, three distinct forms of treatment were sought depending on the perceived cause, the severity of symptoms, and the need of the consumer. Some wanted medicinal products, presumably for stronger relief of sore throat symptoms. Others wanted products with a more sensorial feel, be that cooling or warming drinks or soothing lozenges, while others sought early treatment, perhaps to prevent the condition getting worse. There were also a proportion of respondents who did not seek treatment. This is likely to be reflective of the individual and personal nature of throat discomfort, and how illness is perceived by the respondent.

As described above, however, environmental factors led to symptoms that were considered to be almost comparable in severity to symptoms caused by infection. The low uptake of medicated products among sufferers of some physical or environmental symptoms is, therefore, surprising as these respondents must suffer significant discomfort and reductions in quality of life. If patients seek help from pharmacists and other healthcare professionals, they should recommend the most suitable and effective medicated products to deliver the relief the patient is seeking at that time – treatment should be tailored to manage the patient’s throat discomfort based not only on its cause, but also on the patient’s expectations of treatment. Previous studies have shown that patients who consult their GP with a sore throat are looking for pain relief rather than an antibiotic prescription [15]. There may also be a need to educate sufferers regarding the most effective management of their throat discomfort or irritation as antibiotics may often be inappropriately prescribed for sore throat as a result of patient pressure [16].

Limitations of this study include the different demographic data available across the countries, which makes direct comparisons across the countries difficult. Although, the demographic data are different, they are still representative of each country. Also, the lower internet penetration in Malaysia at the time of the online survey may have led to a bias towards younger age groups, professionals/full-time employees, and city locations.

## Conclusions

In conclusion, these data provide key insights into the feelings and behaviours of consumers suffering with throat irritation. First, not all throat discomfort is the same as demonstrated by the range of perceived causes and the emotional and physical symptoms experienced by respondents. Second, the needs of patients regarding the treatment of throat discomfort differ; therefore, treatment should be tailored by pharmacists not only to suit the cause of the throat discomfort and the severity of symptoms, but also the individual needs of the patient. Pharmacists should advise the most appropriate treatments based on the active ingredients and a range of different treatment options should be provided depending on whether the patient requires rapid, ‘hard-hitting’ relief, sensorial relief, or prevention/protection against symptoms.

## Competing interests

Adrian Shephard is a full-time employee of Reckitt Benckiser and Dilys Addey is a former employee of Boots Healthcare International, now part of Reckitt Benckiser. Reckitt Benckiser are the manufacturers of Strepsils® lozenges for sore throat.

## Authors’ contributions

DA was responsible for research design, identification of appropriate international market research agencies, commissioning of research in all four countries, development of the questionnaire, liaison with the research agencies throughout the course of the project and interpretation of results. DA and AS were both responsible for critical revision and approval of the article.

## Pre-publication history

The pre-publication history for this paper can be accessed here:

http://www.biomedcentral.com/1472-6815/12/9/prepub

## Supplementary Material

Additional file 1 Causes of Throat Irritation & Consumer Attitudes & Behaviour (UK) Draft Questionnaire 2. Click here for file
