# Supplementary material for: Incidence, causes, severity and treatment of throat discomfort: a four-region online questionnaire survey
Source: BMC Ear Nose Throat Disord. 2012 Aug 10;12:9. doi: 10.1186/1472-6815-12-9 (PMC3489867; doi:10.1186/1472-6815-12-9)
Supplement: Additional file 1 — Causes of Throat Irritation & Consumer Attitudes & Behaviour (UK) Draft Questionnaire 2. [file 1472-6815-12-9-S1.doc]

**Causes of Throat Irritation & Consumer Attitudes & Behaviour (UK)**

**Draft Questionnaire 2**

**Screen 1 – Introduction**

Q1 In the last 12 months have you suffered from any of the following conditions, **even if your condition/degree of suffering was only temporary or extremely mild?** (Please select all that apply)

**(DP PLEASE ROTATE LIST)**

Headache

Backache

Throat Irritation or discomfort

Dandruff or itchy/flaky scalp

Sleeplessness

Hayfever

**IF THROAT IRRITATION OR DISCOMFORT NOT MENTIONED, THANK AND CLOSE INTERVIEW (STANDARD TEXT)**

Q2a Thinking about the following possible causes of throat irritation or discomfort, which of the following, if any, have caused you throat irritation/discomfort in the past? (please tick all that apply)

Q2a(i) Since you have mentioned a number of different causes of throat irritation/discomfort, could you please select the five causes that you suffer from most frequently?

Q2b Which has been the most common cause?

(Please select one answer only)

Q2c And what was the cause of the **most recent** throat irritation/discomfort that you experienced?

(Please select one answer only)

(**DP: PLEASE ROTATE LIST)**

Common cold/flu

Other bacterial or viral infection

Hayfever

Specific allergy (excluding hayfever)

Too much talking/shouting/singing

General airborne pollution

Dust or other specific environmental conditions

Smoking

Passive smoking

The morning after drinking too much alcohol

Hot and dry indoor environment

Air conditioning

Sudden changes in temperature

Snoring

Other (please specify)

Q4 How often do you suffer from throat irritation/discomfort as a result of **(insert mentions from Q2a).** Please select one answer only.

**(DP: USE SAME FIVE CAUSES AT Q2a)**

Hardly ever

Only once or twice a year

3-4 times a year

Every month

Every week

More frequently than every week

Q5a Generally, how severe would you rate the degree of throat irritation/discomfort that you experience as a result of **(insert mentions from Q2a)**

Q5b And how severe would you rate the degree of throat irritation/discomfort that you experienced the last time caused by **(insert answer from Q2c)?**

| Very Mild | |  | Very Severe | |
| --- | --- | --- | --- | --- |
| 1 | 2 | 3 | 4 | 5 |

# Q6 Generally, when you have throat irritation/discomfort, which one of these statements most accurately describes how you feel?

#

# (DP PLEASE ROTATE LIST)

# It barely effects me

# I become Intolerant of friends / family / colleagues

# Feel down / sad / depressed

# Feel unsociable

# Frustrated with reduced ability to concentrate

# Frustrated with lack of energy

# Feel sorry for myself

# Frustrated that I’m not in control

# Frustrated that I can’t operate at 100%

# Something else (please specify)

# Q7a Generally, what course of action do you take if you suffer throat irritation/discomfort caused by (insert answers from Q2a)? Please select all that apply.

# (DP PLEASE ROTATE LIST)

# Nothing, just ignore it

# Drink hot drinks such as tea, blackcurrant or lemon drinks

# Drink cold drinks such as water or fruit juice

# Use medicated products such Dequadin, Strepsils etc.

# Use throat sweets such as Tunes, Halls or Lockets

# Use confectionary such as mints or boiled sweets

# Use chewing gum

# Take vitamin tablets or supplements

# Take herbal remedies or other alternative therapies

# Consult my GP or seek/sought other medical advice

# Something else (please specify)

# ASK Q7b IF CONFECTIONARY OR CHEWING GUM USED AT 7a FOR ANY CAUSES

# Q7b Why do you use (answer from Q7a – repeat for both categories if necessary)

# ______________________________________________________________

# ______________________________________________________________

# Q7c Thinking about the last time you experienced throat irritation/discomfort caused by (insert answer from Q2c) what course of action did you take? Please select all that apply.

# Nothing, just ignored it

# Drank hot drinks such as tea, blackcurrant or lemon drinks

# Drank cold drinks water or fruit juice

# Used medicated products such Dequadin, Strepsils etc.

# Used throat sweets such as Tunes, Halls or Lockets

# Used confectionary such as mints or boiled sweets

# Used chewing gum

# Took vitamin tablets or supplements

# Took herbal remedies or other alternative therapies

# Consulted my GP or seek/sought other medical advice

# Did something else (please specify)

# Q7d ONLY ASK Q7d to THOSE WHO ANSWERED ‘NOTHING JUST IGNORED IT’ (CODE 1) AT Q7c

# Why did you not take any action to alleviate your throat irritation/discomfort caused by (insert answer from Q2c)?

# ______________________________________________________________

# ______________________________________________________________

#

# ASK ALL

# Q8a Generally how does your throat feel when you suffer irritation or discomfort as a result of (insert answers from Q2a)? Please select all that apply.

# Q8b Thinking about the last time you experienced throat irritation/discomfort, caused by (INSERT ANSWER FROM Q2c), how did your throat feel?

#

# (DP PLEASE ROTATE LIST)

Dry

Tickly

Scratchy

Husky

Irritated

Hurts to swallow

Painful to talk

Swollen

Inflamed

Burning

Like I've swallowed broken glass/barbed wire

Not sure, depends on cause

# Other (please specify)

#

# Q9 When you have throat irritation/discomfort, which one of these things causes you the most discomfort? Please select one answer only.

# (DP PLEASE ROTATE LIST)

# Eating

# Drinking

# Swallowing

# Talking

# Coughing

# Difficulty sleeping

# Discomfort when breathing

# Smoking

# Not sure depends on cause

# Other (please specify)

# Q10 Thinking about medicated products that might be used to alleviate throat irritation/discomfort, which statement best describes each of the following brands? Please select all that apply.

(Create grid with, Beechams, Benylin, Halls, Lemsip, Lockets, Strepsils & Tunes)

Is the only brand I would ever consider

Would be one of a number of brands I would consider

Would consider using but no strong preference

Would prefer not to use but would consider under some circumstances

I’ve heard of this brand but don’t know much about it

I’ve never heard of this brand

I Would never use this brand

**DP IF MORE THAN ONE BRAND SELECTED FOR FIRST STATEMENT, ASK Q10a.**

Q10a You said that the only brands you would ever consider are (DP INSERT BRANDS FROM STATEMENT 1 AT Q10), please indicate which brand you would consider most………..

# ASK ALL

# Q11a Which products have you ever used or purchased? (MULTIPLE ANSWER)

# ASK Q11b IF MEDICATED PRODUCTS OR THROAT SWEETS PURCHASED AT Q7a

# Q11b Which product/brand do you buy most often? (SINGLE ANSWER ONLY)

# ASK Q11C IF MEDICATED PRODUCTS OR THROAT SWEETS PURCHASED AT Q7c

# I

# Q11c Thinking about the last time you experienced throat irritation/discomfort caused by (insert answer from Q2c) which of the following products did you buy?

#

#

Dequadin

Beechams Throat Plus Lozenges

Benylin Sore Throat Lozenges

Boots Sore Throat Lozenges

Halls Mentholyptus

Halls Soothers

Lemsip Sore Throat Lozenges

Lockets

Strefen

Strepsils

Strepsils Extra

Tunes

Other (please specify)

None

Q12 How strongly do you agree with the following statements that have been made about **(product/brand used most often at Q11b**)? Please select all that apply.

# IF MEDICATED PRODUCTS OR THROAT SWEETS NOT MENTIONED AT 7c SKIP Q12-Q13

Agree Strongly

Agree Slightly

Neither Agree nor Disagree

Disagree Slightly

Disagree Strongly

Don’t Know

**(DP – ORDER OF STATEMENTS TO BE RANDOMISED IN GRID)**

Is very effective at relieving my throat discomfort

Starts working quickly

I can feel it actively relieving my throat

Works by coating my throat

Gives long lasting relief

Works by numbing the discomfort

Comes in a range of pleasant flavours

Is easy to get hold of

Is good value for money

Is more effective than other brands

I can use the product frequently without fear of overdosing

Has convenient packaging

Is suitable for a range of throat problems

Q13 Why did you first start using **(product/brand used most often at Q11b)** Please select all that apply.

**(DP: PLEASE ROTATE LIST)**

My mother/father gave it to me when I was a child

Recommended by pharmacist /doctor

Recommended by friend / family

Influenced of advertising

Because it's a well-known brand

Because of the flavours on offer

# Because it was the only product available at the time

# Not sure/can’t remember

# Other reason (please specify)

# ASK Q14 TO 16 ONLY IF MEDICATED PRODUCTS/THROAT SWEETS USED AT Q7a. ALL OTHERS SKIP TO Q17

Q14 If you use medicated products/throat sweets to alleviate throat irritation/discomfort, do you usually tend to keep a supply at hand?

Yes, I usually have a supply handy

No, I would need to purchase if/when the symptoms arose

Q15 Where do you tend to keep these products?

Medicine cabinet / first aid kit

Bathroom

Kitchen

Handbag/briefcase

Car

Office

Other (please specify)

Q16 During periods of suffering from throat irritation/discomfort, where do you tend to keep these products?

Keep in one place

Carry around for use when needed

We would now like to ask you some further questions to classify the responses you

have previously given into different groups

Q17a Which of the following statements best describes your attitude to illness? Please tick one only.

I live life to the full and accept the consequences (minor illnesses being one of them)

Life is busy and sometimes I might get ill but I just take the necessary medication and get on with it

I take care to stay healthy and try to avoid any type of illness or discomfort

Illness is a real source of anxiety and is very disruptive to my life

Q17b Which of the following best describes your attitude to illness and taking medication?

Please tick one only.

As soon as symptoms appear I take the most appropriate medication (or seek medical advice) as quickly as possible

I tend to see how symptoms develop and if they don’t disappear quickly I take the appropriate medication (or seek medical advice)

I tend to see how symptoms develop, and only take the appropriate medication (or seek medical advice) if the problem becomes really severe

I prefer to avoid medication and just get on with life putting up with discomfort until symptoms disappears naturally

Q18a Are you a smoker?

Yes

No

(**IF YES AT Q18a, ASK Q18b. IF NO AT Q18a SKIP TO Q19)**

Q18b Which of the following best describes your smoking habits?

I rarely smoke/I am a social smoker

I am a moderate smoker

I am a heavy smoker

Q19 Depending upon your occupation or lifestyle, people use their voice to different degrees. For example, a teacher is likely to spend much of the day talking to the class, whereas, talking is less likely to be as important to a train driver. We’d like to understand how much you use you voice, over and above normal conversation (such as making speeches, presentations etc.) in your current job or daily life.

I rarely have to use my voice over and above normal conversation

I use my voice a fair bit

I use my voice a lot

Q20 Which of the following best describes the area in which you live? Please select one only)

City

City suburbs

Small town

Village

Rural/remote area

Q21 Using the grid below, please can you indicate the number of other members in your household which fall into the following age groups:

**(DP: TO INTRODUCE BOX SYSTEM FOR RESPONDENTS TO TYPE IN NUMBERS IN HOUSEHOLD FOR EACH AGE BAND BELOW)**

Children age 0-12

Children age 13-17

Other adults age 18+

Q22 Can you please just confirm your age

|  |  |
| --- | --- |

Q23 Are you?

Male

Female

Q24 Finally, can you please tell me if you agree or disagree with the following list of statements. This will help us better understand your general attitudes to life.

Agree 1

Disagree 2

**DP: RANDOMISE ORDER OF STATEMENTS**

I only buy medicine as a last resort

Minor ailments rarely go away by themselves

Showing pain is not a sign of weakness

I am good at coping with difficult situations

I rarely keep medicines in the house

I usually opt for strongest medicines

I burn the candle at both ends

I am often the life and soul of the party

I live for today

I am careful about what I eat and drink

Medicines help me cope with the side effects of my lifestyle

I sometimes worry about small things

My family rely on me to look after them

The world is becoming a more dangerous place

I like to take decisions quickly

I like to be well informed on health issues

I take a first aid kit on holiday

I like to plan ahead

I don't mind it when people are late

I don't understand disorganised people

If a jobs worth doing, its worth doing well

I don’t worry about my health very much

I hate fuss

I am generally satisfied with my life

I believe in social responsibility

Every problem can be solved if you have a positive attitude

If you are ill just take some medicine

I like to help others

My friends are really important to me

When I get a cold I always seem to suffer badly

I hate being the centre of attention

I believe life is a rat race

I think saving for the future is important

**THANK AND CLOSE – PLEASE USE STANDARD UK PANEL CLOSING STATEMENT**
